# Desmoid tumor in Gardner's Syndrome presented as acute abdomen

**DOI:** 10.1186/1477-7819-4-18

**Published:** 2006-03-28

**Authors:** Andreas Hatzimarkou, Dimitrios Filippou, Vasilios Papadopoulos, Georgios Filippou, Spiros Rizos, Panagiotis Skandalakis

**Affiliations:** 1 1^st ^Department of General Surgery, GP Hospital "Tzaneio", Pireaus, Tzani & Afentouli str. Pireaus, Athens, Greece; 2 Department of Internal Medicine, Medical School, Democrition University of Thrace, Alexandroupoli, Greece; 3 1^st ^Department of Surgery, Medical School, University of Athens, Mikras Asias 75, 11527 Goudi, Athens, Greece

## Abstract

**Background:**

Gardner's syndrome can occasionally be complicated with intra-abdominal desmoid tumor. These tumors usually remain asymptomatic but can exhibit symptoms due to intestinal, vascular and ureteral compression and obstruction.

**Case presentation:**

A rare case of a 41-year-old male patient with Gardner's syndrome complicated with intra-abdominal desmoid tumor, which first presented as acute abdomen, is presented.

**Conclusion:**

Extra-abdominal manifestations of Gardner's syndrome along with a palpable abdominal mass would raise suspicion for the presence of a desmoid tumor in the majority of cases. In life-threatening cases, surgical treatment should be considered as a palliative approach, though the extent of excision remains debatable

## Introduction

Gardner's syndrome is considered to be a variant of familial adenomatous polyposis, as both entities are caused by mutations in the APC gene, 20% of which are *de novo *[1]. Gardner's syndrome is characterized by colorectal cancer in early adult life secondary to extensive adenomatous polyps of the colon. Polyps also develop in the upper gastrointestinal tract and malignancies may occur in other sites including the brain and the thyroid. Moreover, osteomatosis of the skull and the mandible, sebaceous cysts, and cutaneous and subcutaneous fibromas are additional characteristics [2].

Hereditary desmoid disease has also been found to be caused, at least in some cases, by mutation in the APC gene [3], whereas mutations in the CTNNB1 gene, responsible for the production of beta-catenin, have also been observed in somatic desmoid tumors [4]. Nevertheless, the partially common genetic background between hereditary desmoid disease and Gardner's syndrome has been clinically suspected long ago as 8% of patients with Gardner's syndrome had been complicated by intra-abdominal desmoid tumors [5,6].

Desmoid tumors are benign and are characterized by fibroblastic proliferation of fascial and musculoaponeurotic origin. They usually remain asymptomatic for a long time before symptoms of intestinal, vascular and ureteral compression and obstruction evolve. They may cause obstructive jaundice or bowel obstruction and fistulization. A rare manifestation involves neural paralysis (quadriceps paralysis or neurogenic bladder) due to compression [7,8]. The role of computed tomography and magnetic resonance imaging in the assessment of diagnosis is crucial [9-12].

## Case presentation

A 41-year-old male was admitted to the Emergency Department complaining for acute sharp abdominal pain followed by abdominal distention, nausea, vomiting and bloody stools. The patient was a heavy smoker (70 cig/24 h) and reported mild abdominal distention during the last month and an episode of anal bleeding 4 years ago, which had been attributed to hemorrhoids. His mother died after surgical operation for multiple polyposis coli.

The physical examination revealed abdominal distention, hypoactive bowel sounds, and a large palpable intra-abdominal mass with peritoneal signs. A palpable osteomatosis of the right mandible, multiple sebaceous cysts and subcutaneous fibromas of the trunk and head were observed. The absence of intestinal sounds, the distention and the abdominal contraction suggested acute abdomen. The blood analysis suggested leukocytosis (WBC 18110/μL out of which 94% were neutrophils) and anemia (Hct 29.3% and Hb 7.7 g/dl).

**Figure 1:**
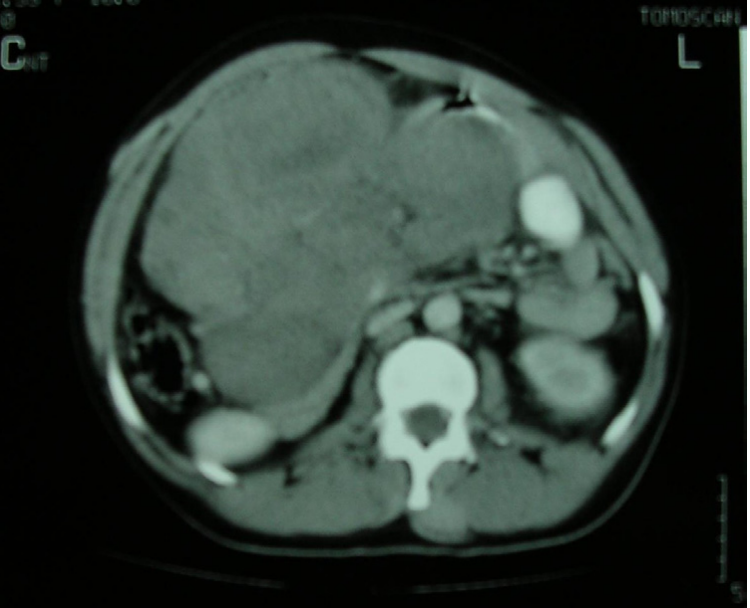
CT scan of the abdomen with contrast media reveals a large-size intra-abdominal mass displacing the adjacent structures. In the same scan a suspicious lesion is identified in the right adrenal. In some rare cases desmoid tumors may co-exist with adrenal or thyroid carcinomas and adrenal adenomas.

Abdominal ultrasound revealed a large mass with necrotic lesions. Triplex study of the mass indicated decreased internal circulation with measurable velocity. An abdominal CT scan that performed after oral gastrographin administration and IV contrast medium infusion, revealed a large intra-abdominal neoplastic mass consisting of tissues of variable density. The mass was extended from the pancreas to the pelvic entrance (figure 1). The tumor displaced the adjacent visceral structures. Other findings include a small diaphragmatic hernia, and a hypodense enlargement of the right adrenal due to adrenal adenoma. Sigmoidoscopy up to 20 cm revealed multiple polyps.

**Figure 2:**
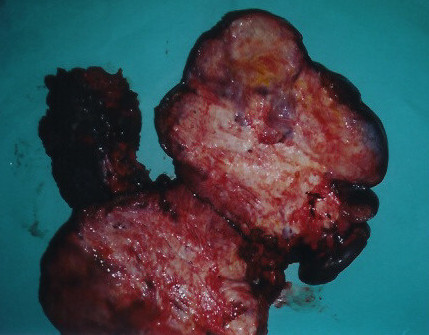
The specimen opened after it had been removed. The operation included tumor wide resection, in combination with right colon resection, partial extended enterectomy and right adrenalectomy.

The patient was surgically treated. After entering the abdominal cavity a large solid neoplasmatic mass which invaded parts of the small and large intestine was recognized. The abdominal tumor was resected, along with the involved parts of the small intestine and the right colon (Figure 2). The excised right colon revealed numerous small and large polyps, suggesting a background of familial polyposis. The adrenal adenoma was also resected; cholecystectomy was carried out due to co-existing cholelithiasis in order to prevent biliary obstruction.

Postoperative ophthalmologic examination of the fundus, revealed the characteristic black spots of congenital hypertrophy of the retinal pigment epithelium typical of Gardner syndrome. Full length colonoscopy was scheduled after patient's discharge. Unfortunately the patient died three months later from acute myocardial infarction. We failed to identify clinically any only obvious risk factors for the subsequent AMI except smoking.

The histological examination of the specimen revealed desmoid tumor. Further cytogenetic studies along with APC mutation analysis confirmed the diagnosis. Mutation was identified in exon 9 and codon 1444.

Extra-abdominal manifestations of Gardner's syndrome along with a palpable abdominal mass would raise suspicion for the presence of a desmoid tumor in the majority of cases. Computed tomography would help to establish the diagnosis [9-12]. The patient was discharged the 12^th ^postoperative day.

## Discussion

Gardner's syndrome consists of extensive adenomatous polyps of the colon, sebaceous cysts, osteomatosis of the skull and the mandible, and cutaneous and subcutaneous fibromas. In some cases this entity is complicated with other malignancies to include periampullary neoplasms and intra-abdominal desmoid tumor. Some authors described a rare co-existence with small bowel adenomas, carcinomas of the thyroid and adrenal gland and hepatoblastomas. The incidence of adrenal adenomas in patients with Gardner syndrome is yet unidentified, as relevant literature is inadequate [12,13].

Intra-abdominal desmoid tumors remain asymptomatic for long periods, and the initial symptoms are usually due to intestinal, vascular or ureteric compression and obstruction, while in rare cases neural paralysis or fistula may be the first sign [14]. Once detected, desmoid tumors should alert the doctor to collect a detailed family history and to perform a clinical examination targeted to colon carcinoma or polyps. Truly, in our patient, the presence of multiple polyps in recto-sigmoidoscopy suggested the diagnosis of familial polyposis coli complicated with the desmoid tumor.

The unique feature of our case is that the prevailing symptom of the tumor was the acute abdomen. Although the diagnosis of Gardner's syndrome was evident due to the presence of various clinical signs suggestive of the disease, the etiology of acute abdomen in our patient remained unclear. Intestinal perforation might be an explanation, but the surgical procedure provided no proof for this speculation. As no similar reports in the literature were identified, this case report is of special interest.

CT scan contributes not only in the diagnosis of the lesion but also in the definition of the extension in the adjacent visceral structures. A possible disadvantage of the CT scan is the inability to distinguish a desmoid tumor from a soft tissue sarcoma. The differential diagnosis of the tumor characteristics can be supported by MRI, although this study cannot assess the margin of the lesion. Recent data suggest that preoperative performance of both CT and MRI may be helpful for accurate diagnosis as well as assessment of the disease extent [9,10].

Though they are not cancer and do not metastasize, desmoids can cause significant morbidity and occasionally death through local/regional invasion of critical structures [14]. Nevertheless, their rarity along with their variability in the clinical course prevented surgeons from the establishment of guidelines.

In general, surgery is not indicated in patients with intraabdominal desmoid tumors and should be reserved for life threatening complications since surgery (including R1, R2 and debulking) is associated with high recurrence rates (over 65%) and may involve major small bowel resection with its consequences. In these patients medical treatment (sulindac, tamoxifen), chemotherapy (doxorubicin, dacarbazin) and radiotherapy or combination of them can result tumor remission [15].

In life-threatening cases, surgical treatment should be considered as a palliative approach, though the extent of excision remains debatable [16]. Some authors support a more conservative resection, while others suggest aggressive resection with uninvolved margin. We believe that wide resection in disease free margins is an important determinant of a successful outcome, decreasing the incidence of postoperative recurrence [17,18].

The kind and extent of resection are determined by the location of the tumor and the adjacent structures that are involved. In our case we tried to achieve an adequate resection margin, thus we excised the part of the small and large intestine that was involved in the lesion. As a suspicious lesion in the right adrenal was identified in the CT scan, colectomy, lumpectomy and enterectomy was combined with right adrenalectomy; this decision was supported by the fact that abdominal desmoid tumors might co-exist with adrenal carcinoma. This combined procedure leads to excision of the lesion in adequate macroscopic and microscopic free of disease margins.

The role of adjuvant radiotherapy and chemotherapy in the treatment of desmoid tumors still remains unclear. Some authors believe that radiotherapy contributes in the local control of the disease [19]. Recent data about the role of adjuvant chemotherapy are obscure and disappointing [20]. In our case no additional adjuvant therapy administrated due to the sudden death of the patient soon after his discharge [14,18,21]

## Conclusion

Gardner's syndrome should be suspected in patients with osteomatosis, multiple sebaceous cysts and cutaneous and subcutaneous fibromas. Although the appropriate treatment of intra-abdominal tumors remains unclear we believe that wide resection in adequate margin offer the best alternative for local control of the tumor.

## Competing interests

The author(s) declare that they have no competing interests.

## Authors' contributions

**HA**: Had made substantial contributions to conception and the patient's treatment. **FD**: Collected the data and wrote the manuscript. **PV**: Corrected the manuscript. **FG**: Collected the data and contributed to the manuscript writing. **RS**: Corrected and approved the manuscript and participate to the patient's treatment. **SP**: Contributed to the conception, corrected and approved the manuscript.

All authors read and approved the final manuscript.
